# Outcomes of Post-Operative Treatment with Concurrent Chemoradiotherapy (CRT) in High-Risk Resected Oral Cavity Squamous Cell Carcinoma (OCSCC): A Multi-Institutional Collaboration

**DOI:** 10.3390/curroncol28040221

**Published:** 2021-06-30

**Authors:** Arslan Babar, Neil M. Woody, Ahmed I. Ghanem, Jillian Tsai, Neal E. Dunlap, Matthew Schymick, Howard Y. Liu, Brian B. Burkey, Eric D. Lamarre, Jamie A. Ku, Joseph Scharpf, Brandon L. Prendes, Nikhil P. Joshi, Jimmy J. Caudell, Farzan Siddiqui, Sandro V. Porceddu, Nancy Lee, Larisa Schwartzman, Shlomo A. Koyfman, David J. Adelstein, Jessica L. Geiger

**Affiliations:** 1 Department of Internal Medicine, Cleveland Clinic Foundation, Cleveland, OH 44195, USA; babara@ccf.org; 2 Department of Radiation Oncology, Cleveland Clinic Taussig Cancer Institute, Cleveland, OH 44195, USA; woodyn@ccf.org (N.M.W.); dr.nikhil.j@gmail.com (N.P.J.); KOYFMAS@ccf.org (S.A.K.); 3 Department of Radiation Oncology, Henry Ford Cancer Institute, Detroit, MI 48202, USA; AGHANEM1@hfhs.org (A.I.G.); mschymi1@hfhs.org (M.S.); FSIDDIQ2@hfhs.org (F.S.); 4 Alexandria Clinical Oncology Department, Alexandria University, Alexandria 00203, Egypt; 5 Department of Radiation Oncology, Memorial Sloan Kettering Cancer Center, New York, NY 10065, USA; tsaic@mskcc.org (J.T.); leen2@mskcc.org (N.L.); 6 Department of Radiation Oncology, University of Louisville Hospital, Louisville, KY 40202, USA; neal.dunlap@louisville.edu (N.E.D.); sandro.porceddu@health.qld.gov.au (S.V.P.); 7 Department of Radiation Oncology, Princess Alexandra Hospital, Brisbane, QLD 4102, Australia; Howard.Liu@health.qld.gov.au; 8 Head and Neck Institute, Cleveland Clinic Foundation, Cleveland, OH 44195, USA; BURKEYB1@ccf.org (B.B.B.); lamarre@ccf.org (E.D.L.); KUJ@ccf.org (J.A.K.); SCHARPJ@ccf.org (J.S.); PRENDEB@ccf.org (B.L.P.); 9 Department of Radiation Oncology, H. Lee Moffitt Cancer Center and Research Institute, Tampa, FL 33612, USA; Jimmy.Caudell@moffitt.org; 10 Department of Hematology and Medical Oncology, Cleveland Clinic Taussig Cancer Institute, Cleveland, OH 44195, USA; SCHWARL@ccf.org (L.S.); ADELSTD@ccf.org (D.J.A.)

**Keywords:** high risk oral cavity cancer, oral cavity squamous cell cancer, chemoradiation, cisplatin, cumulative cisplatin dose, cisplatin schedule

## Abstract

Adjuvant chemoradiation (CRT), with high-dose cisplatin remains standard treatment for oral cavity squamous cell carcinoma (OCSCC) with high-risk pathologic features. We evaluated outcomes associated with different cisplatin dosing and schedules, concurrent with radiation (RT), and the effect of cumulative dosing of cisplatin. An IRB-approved collaborative database of patients (pts) with primary OCSCC (Stage I–IVB AJCC 7th edition) treated with primary surgical resection between January 2005 and January 2015, with or without adjuvant therapy, was established from six academic institutions. Patients were categorized by cisplatin dose and schedule, and resultant groups compared for demographic data, pathologic features, and outcomes by statistical analysis to determine disease free survival (DFS) and freedom from metastatic disease (DM). From a total sample size of 1282 pts, 196 pts were identified with high-risk features who were treated with adjuvant CRT. Administration schedule of cisplatin was not significantly associated with DFS. On multivariate (MVA), DFS was significantly better in patients without perineural invasion (PNI) and in those receiving ≥200 mg/m^2^ cisplatin dose (*p* < 0.001 and 0.007). Median DFS, by cisplatin dose, was 10.5 (<200 mg/m^2^) vs. 20.8 months (≥200 mg/m^2^). Our analysis demonstrated cumulative cisplatin dose ≥200 mg/m^2^ was associated with improved DFS in high-risk resected OCSCC pts.

## 1. Introduction

In 2004, two randomized controlled trials, Radiation Therapy Oncology Group (RTOG) 9501 and European Organization for Research and Treatment of Cancer (EORTC) 22931, reported improved outcomes when chemotherapy was added to post-operative radiotherapy (PORT) in high-risk resected head and neck squamous cell carcinoma (HNSCC) [1,2,3]. A combined analysis demonstrated patients with high-risk features, defined as either positive surgical margins (SM+) or extracapsular extension (ENE), benefitted the most from the addition of cisplatin [4]. These results established the standard of care treatment for resected high-risk HNSCC with high-dose cisplatin (100 mg/m^2^) every three weeks, administered concurrently with radiation (RT) [5]. However, cisplatin is highly emetogenic, nephrotoxic, ototoxic, and myelosuppresive [6,7], precluding such use in patients with significant medical co-morbidities or poor social support. Many institutions and oncologists have embraced different cisplatin schedules to deal with the toxicity, albeit with a lack of head-to-head prospective evidence supporting such alternatives as equivalent substitutes for the standard high-dose cisplatin [7]. Several studies using a weekly dosed cisplatin 40 mg/m^2^ have been reported [8,9,10,11]. Other dosing schedules include 30 mg/m^2^ weekly [12] or 50 mg (fixed dose) weekly [13]. Daily low-dose cisplatin at 6 mg/m^2^ with RT has also been found feasible [14]. All of these regimens are administered concurrently with RT and are used in both post-operative and definitive settings.

It remains unclear if dosing schedule or total cumulative dose, during the concurrent radiation, is more important with respect to outcomes. The present study investigated cumulative cisplatin dosing and effects of cisplatin administration schedule on disease free survival (DFS), overall survival (OS), locoregional control (LRC), and freedom from metastatic disease (DM) in patients with high-risk resected oral cavity squamous cell carcinoma (OCSCC).

## 2. Materials and Methods

Patients were identified from an IRB-approved multi-institutional collaborative database of primary OCSCC (Stage I–IVB AJCC 7th edition) [15] from six academic institutions treated with primary surgical resection with or without adjuvant therapy between January 2005 and January 2015. Patients who demonstrated high-risk features of ENE and/or SM+, and who went on to receive adjuvant concurrent cisplatin, were included in this analysis and were categorized by cisplatin dose received. The resultant groups were compared for demographic data, multiple pathologic features in addition to ENE, margin assessment, and outcomes assessed by t-test and Chi-squared tests. Kaplan-Meier curves, log-rank *p*-values, and multivariate analysis (MVA) were used to determine DFS and DM. Variables with *p* ≤ 0.05 on univariable Cox hazard analysis were incorporated into MVA to assess independent predictors of DFS and DM.

Variables used for MVA for DFS were perineural invasion (PNI), ENE, and cumulative cisplatin dose. Variables used for MVA for DM were PNI and cumulative cisplatin dose. DFS was defined as the time from initial diagnosis to tumor recurrence. OS was defined as time from initial diagnosis until death from any cause.

## 3. Results

### 3.1. Patient Characteristics

**Figure 1:**
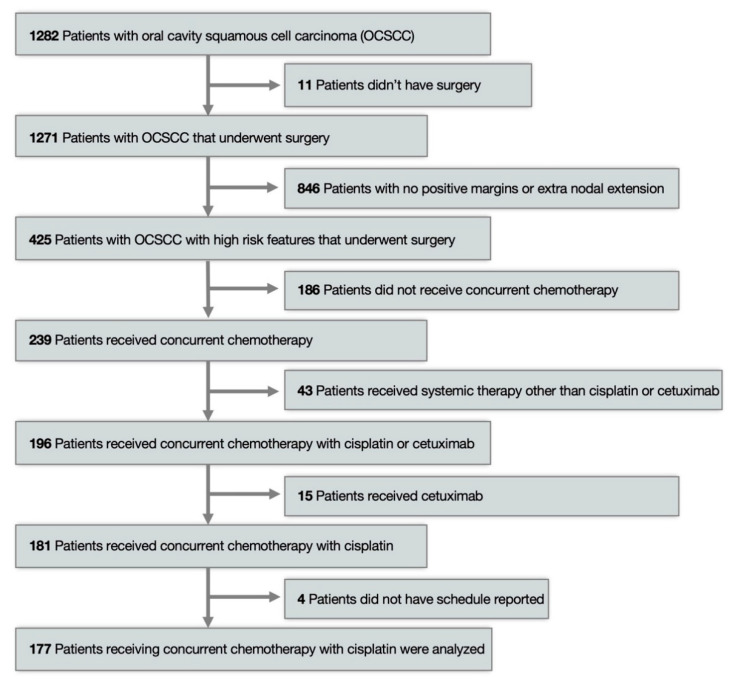
Patient Selection.

From a total sample size of 1282 OCSCC patients, we identified 196 (15.3%) patients with high-risk features who were treated with concurrent chemoradiation (CRT) with cisplatin or cetuximab (Figure 1). Out of 196 patients, 181 received concurrent chemotherapy with cisplatin. Median age was 56 years; 63.3% of patients were male, 81.1% were Caucasian, 71.3% had a median 30 pack-year smoking history, 28.7% did not have a smoking history. All patients had high-risk features: 35.7% had SM+, and 83.9% had ENE. Regarding histology, 64.8% of tumors were moderately differentiated, 31.1% were poorly differentiated, and 4.1% were well differentiated. Staging information was collected: 14.3% were T1, 33.2% were T2, 9.7% were T3, and 42.8% were T4a or T4b; 73% were N2b or greater by AJCC 7th edition [15]. Patient characteristics are summarized in Table 1.

### 3.2. Treatment

All patients underwent surgical resection; 96.9% patients underwent surgical resection with lymph node dissection, and 3.1% underwent surgical resection without lymph node dissection.

A total of 181 patients (92.3%) of the cohort received concurrent cisplatin and radiation in this analysis: 67.4% (122 pts) received high-dose cisplatin, and 30.4% (55 pts) received weekly cisplatin 40 mg/m^2^. In four patients, the cisplatin schedule was not identified, and 15 patients received cetuximab. Median dose of RT delivered was 66 Gray (Gy).

The estimated 3-year and 5-year overall survival (OS) rates for the whole cohort were 51% and 45.7%, respectively. On univariate analysis, OS was significantly worse in patients with PNI (HR: 1.9, 95% CI 1.2 to 2.9, *p* = 0.003) and with lymphovascular space invasion (LVSI) (HR: 1.7, 95% CI 1.1 to 2.4, *p* = 0.003). The OS was better with the radiation dose (per 10 Gy) received (HR: 0.75, 95% CI 0.631 to 0.89, *p* = 0.002). On univariate analysis, LRC was worse with the presence of PNI (HR: 2.09, 95% CI 1.2 to 3.6, *p* = 0.008). DM was worse with PNI (HR: 2.6, 95% CI 1.3 to 5.3, *p* = 0.005), LVSI (HR: 2.5, 95% CI 1.4 to 4.6, *p* = 0.002), and ECE (HR: 2.9, 95% CI 1.1 to 8.3, *p* = 0.03). DFS was significantly better with higher cisplatin dose received (HR: 0.4, 95% CI 0.91 to 0.99, *p* = 0.013) and higher RT dose delivered (HR: 0.9, 95% CI 0.95 to 0.99, *p* = 0.008), and worse with PNI (HR: 1.9, 95% CI 1.2 to 2.8, *p* = 0.002) and LVSI (HR: 1.4, 95% CI 1.1 to 2.1 *p* = 0.031).

**Figure 2:**
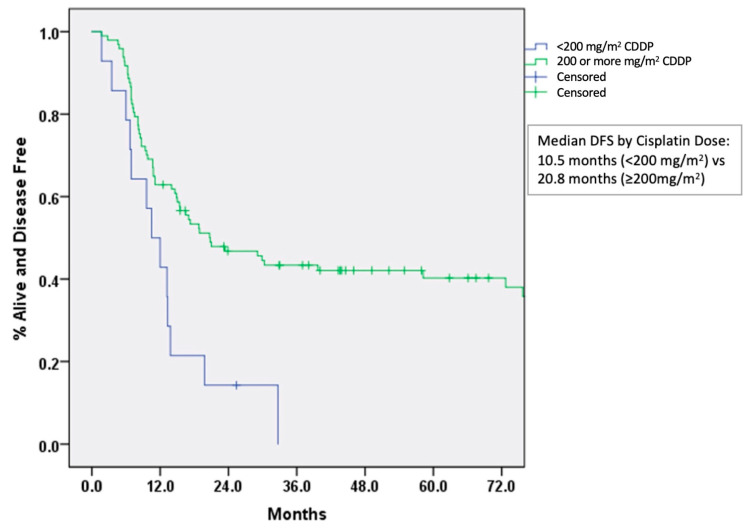
Disease free survival by dose of concurrent cisplatin. Abbreviations: CDDP, Cisplatin; DFS, Disease free survival.

On MVA, DFS was significantly better with higher cisplatin dose (HR: 0.95, 95% CI 0.914 to 0.99 per 100 mg/m^2^ increase in cisplatin, *p* = 0.007) and absence of PNI (HR: 3.1, 95% CI 1.7 to 5.5, *p* < 0.001) (Table 2). Median DFS was significantly better with higher cisplatin cumulative dose: 10.5 months for <200 mg/m^2^ vs. 20.8 months for ≥200 mg/m^2^ (*p* = 0.013) (Figure 2). Median OS by cisplatin cumulative dose was not significantly different (*p* = 0.187). DM was significantly higher in patients with PNI (HR: 2.7, 95% CI 1.1 to 6.5, *p* = 0.031). There was a trend towards improved outcomes with higher cumulative cisplatin dose delivered: OS (*p* = 0.187), LRC (*p* = 0.131), and DM (*p* = 0.084). Cisplatin administration schedule (weekly vs. every 3 weeks) was not associated with significant effect on DFS (HR: 0.618, 95% CI 0.59 to 1.36). 

## 4. Discussion

High-dose cisplatin added to adjuvant RT is the standard chemotherapy for high-risk resected OCSCC, with a benefit in survival outcomes compared with adjuvant RT alone [1,3,4]. However, concern for toxicities has led to questions regarding alternative dosing strategies to maintain optimal survival outcomes while mitigating such adverse effects. Retrospective data support use of both schedules in this disease [16,17] and prospective data demonstrate non-inferiority of weekly cisplatin in high-risk locally advanced HNSCC [18].

Similarly, our data, evaluating one of the largest cohorts for OCSCC, treated using modern modalities, demonstrate no difference in DFS with respect to cisplatin administration schedule (weekly vs. once every three weeks). Total cumulative dose is more important, with patients receiving ≥200 mg/m^2^ of cisplatin demonstrating improved DFS in our cohort of high-risk resected OCSCC patients. Though not statistically significant, a trend was also noticed towards improved LRC and OS in patients who received a ≥200 mg/m^2^ cumulative dose.

These data are reassuring, as high-dose cisplatin can be very toxic with a multitude of short- and long-term side effects, which may limit its use in vulnerable patients [18,19]. Weekly cisplatin may be a feasible alternative for patients unable to tolerate the three-weekly high-dose regimen [9,10,12,13].

Prospective data, comparing cisplatin dosing schedules, have also been published [18,19]. A phase-III randomized non-inferiority trial, published in 2017, reported data supporting three-week cisplatin [19]. An overwhelming majority of patients were oral cavity (87%) and received adjuvant CRT (total 93%). Two-year LRC was significantly better in the cohort that received high-dose three-weekly cisplatin (73.1 vs. 58.5%, *p* = 0.014). Of note, the median cumulative cisplatin dose in the weekly cohort was 210 mg/m^2^ compared with 300 mg/m^2^ in the three-weekly cohort. Grade 3, or higher, toxicities occurred more frequently in the high-dose cisplatin group (84.6% vs. 71.6%, *p* = 0.006). Outcomes from retrospective and prospective studies, comparing weekly versus three-weekly cisplatin, are summarized in Table 3, including the recently published Japan Clinical Oncology Group study (JCOG1008) [18] demonstrating non-inferiority of weekly cisplatin.

While there is new support for alternative cisplatin dosing schedules, there is a paucity of literature on the optimal cumulative dose of cisplatin, but some studies have suggested that it may have a more significant impact on overall survival than the cisplatin dosing schedule used [23,24].

One systematic review, based on six phase-III trials of definitive CRT, found a statistically significant association between OS and cumulative cisplatin dose (*p* = 0.027) [25]. This review concluded that a survival benefit was shown at 140–200 mg/m^2^, that higher cumulative dose resulted in greater benefit, and that a cumulative dose of at least 200 mg/m^2^ cisplatin is recommended.

In the definitive setting, two retrospective studies compared high-dose and low-dose cisplatin and found no significant difference in OS between the two groups [16,26]. However, one of the retrospective studies found that, when analysis was limited to human papilloma virus–negative (HPV-) locally advanced head and neck cancers, a cumulative dose of greater than 200 mg/m^2^ had a statistically significant effect on OS [26]. Our study, similarly, found a trend toward OS improvement in resected oral cavity cancers.

A recent multi-center retrospective study done in Switzerland included 314 patients with advanced HNSCC, including 18.3% oral cavity, treated with combined CRT in the adjuvant (35.1%) and definitive (64.9%) settings, at three different centers between 2008 and 2015 [22]. The study failed to find a significant association between cisplatin dose of ≥200 mg/m^2^ and improved OS or progression free survival (PFS). It did, however, show that more patients treated with the high-dose three-weekly regimen were able to receive a dose of ≥200 mg/m^2^.

Overall, these studies suggest that a cumulative dose of ≥200 mg/m^2^ cisplatin confers an OS benefit compared to <200 mg/m^2^; however, it is unclear if further dose increase confers further OS benefit.

In our study, we evaluated outcomes associated with different cisplatin schedules, concurrent with radiotherapy, and the effect of cumulative dosing of cisplatin. Our findings largely align with the general trends observed above, indicating the importance of a cumulative dose of ≥200 mg/m^2^ cisplatin, though with varying degrees of significance for the variables measuring treatment success (DFS, OS, LRC, and DM). Most significantly, our study demonstrated that a higher dose of cisplatin significantly improved DFS, which has not been reported in previous studies.

Furthermore, our results also indicated a trend associating higher cisplatin dose with improved OS (*p* = 0.187). However, we did not observe a statistically significant improvement in OS with the higher dose.

Multiple studies have shown that the presence of PNI in HNSCC has been associated with worse survival outcomes [27,28,29]. Similarly our study also showed that the presence of PNI was associated with worse OS, DFS, LRC, and DM.

Selection bias serves as a limitation on this study. Conceivably, patients selected for high-dose chemotherapy are likely younger and in better health, able to undergo more rigorous treatment. In this case, age and health status could have acted as confounding variables.

## 5. Conclusions

Based on our multi-institution collaborative cohort of retrospective data, we found that a dose of ≥200 mg/m^2^ of cisplatin had a significant impact on DFS in high-risk resected OCSCC. Our study is one of the largest of its kind and one of the first to report on the association between cumulative dosing and survival outcomes in the adjuvant setting. Cisplatin administration schedule (weekly vs. every 3 weeks) was not associated with significant effect on DFS. Our study also showed that the presence of PNI was associated with worse survival outcomes. A randomized controlled trial would be required to better define the effects of cumulative dose on survival outcomes in the adjuvant setting.

## Figures and Tables

**Table 1:** Patient characteristics.

Characteristic	*n*	%
Sex		
Female	72	36.7
Male	124	63.3
Race		
Black	14	7.1
White	159	81.1
Other	23	11.7
Tobacco use (1 unknown)		
Yes	139	71.3
No	56	28.7
Tumor site		
Tongue	103	52.6
Floor of mouth	34	17.3
Gingiva	19	9.7
Retromolar trigone (RMT)	11	5.6
Buccal	17	8.7
Other	12	6.1
Margin status		
Positive	70	35.7
Negative	126	64.3
Extranodal extension (ENE) (3 unknown)		
Yes	162	83.9
No	31	16.1
Perineural invasion (PNI) (1 unknown)		
Yes	128	65.6
No	67	34.4
Lymphovascular space invasion (LVSI) (3 unknown)		
Yes	96	49.7
No	97	50.3
Grade		
Well differentiated	8	4.1
Moderately differentiated	127	64.8
Poorly differentiated	61	31.1
AJCC 7 pathologic T		
T1	28	14.3
T2	65	33.2
T3	19	9.7
T4a/T4b	84	42.8
AJCC 7 pathologic N		
N0/no nodal dissection	15	7.7
N1/N2a	37	18.8
N2b	115	58.7
N2c	28	14.3
N3	1	0.5
Systemic therapy		
Cisplatin	181	92.3
Schedule:		
Q 3 week	122	67.4
Q week	55	30.4
Unknown	4	2.2
Non-cisplatin-based chemotherapy (cetuximab)	15	7.7
Cisplatin dose received: Median: 200 mg/m^2^ (range 80–300)		
≥200 mg/m^2^	158	87.4
<200 mg/m^2^	23	12.6
Radiation dose received: Median 66 Gy (range 10–76)		

**Table 2:** Multivariate disease-free survival (DFS).

Treatment Characteristics	Hazard Ratio (HR)	95% Confidence Interval (CI)	*p*-Value
Cisplatin (CDDP) dose received (per 100 mg/m^2^)	0.951	0.914–0.990	0.007
Perineural invasion (PNI)	3.077	1.706–5.525	<0.001

**Table 3:** Characteristics of retrospective and prospective studies comparing weekly versus three-weekly cisplatin. Abbreviations used are as follows: CI, confidence interval; HPV, human papillomavirus; HR, hazard ratio; LRC, locoregional control; NR, not reported; OS, overall survival; PFS, progression free survival; RFS, recurrence free survival.

Study	Therapy Intent	Study Arms	Number of Patients	Oral Cavity	Median or Cumulative Cisplatin Dose (mg/m^2^)	Outcomes: Weekly vs. 3-Weekly	Conclusions	Cumulative Dose Outcomes for OS
Espeli et al. 2012 [8]	Adjuvant (44.7%) Definitive (52.3%)	Weekly (40 mg/m^2^) 3-weekly (100 mg/m^2^)	Total: 94 Weekly: 40 (42.6%) 3-weekly: 54 (57.4%)	Total: 33 (35%) Weekly: 15 (37.5%) 3-weekly: 18 (33.3%)	Weekly: 186 mg/m^2^ 3-weekly: 232 mg/m^2^ (*p* = 0.0002)	Median OS at 2.8 years: 1.9 years vs. 4.3 years (*p* = 0.041) Median PFS: 1.5 years vs. 2.1 years (*p* = 0.47)	Improved OS with 3-weekly cisplatin Increased chronic renal toxicity with 3-weekly cisplatin (*p* = 0.04)	>240 mg/m^2^ cisplatin associated with better OS
Geiger et al. 2014 [20]	Adjuvant	Weekly (30 mg/m^2^) 3-weekly (100 mg/m^2^)	Total: 104 Weekly: 53 (50.9%) 3-weekly: 51 (49%)	Total: 26 (25%) Weekly: 16 (30%) 3-weekly: 10 (20%)	Weekly: 150 mg/m^2^ 3-weekly: 200 mg/m^2^ (*p* = 0.01)	3-year OS: 75% vs. 84% (*p* = 0.30) 3-year RFS: 74% vs. 71% (*p* = 0.95)	Trend towards improved survival with high-dose cisplatin in HPV/p16-positive oropharynx cancer	NR
Rades et al. 2016 [21]	Definitive	Weekly (30–40 mg/m^2^) 3-weekly (100 mg/m^2^)	Total: 133 Weekly: 75 (56.3%) 3-weekly: 58 (43.7%)	Total: 15 (11%) Weekly: 8 (11%) 3-weekly: 7 (12%)	NR	Improved LRC [HR] 1.57; *p* = 0.008) and OS in three-weekly (HR 1.33; *p* = 0.023).	Improved OS and LRC with 3-weekly cisplatin Increased hematotoxicity, renal failure, and pneumonia/sepsis with 3-weekly cisplatin	NR
Helfenstein et al. 2019 [22]	Adjuvant Definitive	Weekly (40–50 mg/m^2^) 3-weekly (100 mg/m^2^)	Total: 314 Weekly: 187 (60.0%) 3-weekly: 127 (40.4%)	Total: 57 (18.3%) Weekly: 27 (14.5%) 3-weekly: 30 (23.8%)	Weekly: 160 mg/m^2^ 3-weekly: 200 mg/m^2^ (*p* = 0.001)	No difference in survival outcomes.	Higher number of patients received cumulative dose >200 mg/m^2^, 75.6% vs. 47.1% (*p* < 0.001) Higher acute renal toxicity with 3-weekly cisplatin	No difference in OS seen with a cumulative dose of >200 mg/m^2^
Bauml et al. 2019 [16]	Definitive	Weekly (40 mg/m^2^) 3-weekly (100 mg/m^2^)	Total: 2901 Weekly: 701 (24.1%) 3-weekly: 2200 (75.9%)	Total: 183 (6.3%) Weekly: 55 (30%) 3-weekly: 128 (70%)	Weekly: 145 mg/m^2^ 3-weekly: 215 mg/m^2^	No difference in survival outcomes.	Higher acute renal toxicity, neutropenia, dehydration/electrolyte imbalance, and hearing loss with 3-weekly cisplatin	NR
Mohamed et al. 2019 [17] 39 studies included in the comparative analysis.	Definitive	Weekly (40 mg/m^2^) 3-weekly (100 mg/m^2^)	Total: 3668 Weekly: 1186 (32%) 3-weekly: 2482 (67%)	NR	Weekly: 200 mg/m^2^ 3-weekly: 300 mg/m^2^	Similar OS at 2 years: 74% vs. 67% (*p* = 0.67). Similar LRC: 58% vs. 61% (*p* = 0.7) Similar 2-year PFS: 69% vs. 62% (*p* = 0.9)	Weekly cisplatin comparable in efficacy and safety to 3-weekly cisplatin	NR
Noronha et al. 2017 [19]	Adjuvant (93%) Definitive (7%)	Weekly (30 mg/m^2^) 3-weekly (100 mg/m^2^)	Total: 300 Weekly: 150 3-weekly: 150	Oral cavity: 262 (87%) Weekly: 136 3-weekly: 126	Weekly: 180–200 mg/m^2^ 3-weekly: 300 mg/m^2^	Trend towards better OS in 3-weekly. Median OS 39.5 months in weekly. Median OS not reached in 3-weekly. HR (1.14 [95% CI, 0.79 to 1.65]; *p* = 0.48). LRC better in 3-weekly vs. weekly: 73.1% vs. 58.5%, (*p* = 0.014)	Better LRC in 3-weekly vs. weekly Higher grade-3 toxicities in 3-weekly vs. weekly, 84.6% vs. 71.6% (*p* = 0.006)	NR
Kunieda et al. 2014 [18] Phase II/III trial (JCOG1008)	Adjuvant	Weekly (40 mg/m^2^) 3-weekly (100 mg/m^2^)	Total: 261 Weekly: 129 (49.5%) 3-weekly: 132 (50.5%)	NR	Weekly: 239 mg/m^2^ 3-weekly: 280 mg/m^2^	3-year OS in 3-weekly vs. weekly, 59.1% vs. 71.5% [HR, 0.69 (99.1% CI, 0.374–1.273 [<1.32] *p* for non-inferiority = 0.00272 [<0.00433]	Weekly cisplatin is non-inferior to 3-weekly cisplatin. Higher kidney injury, neutropenia, and mucositis in 3-weekly arm	NR

## Data Availability

The data presented in this study are available on request from the corresponding author.
